# Questionable Advertisements

**Published:** 1857-07

**Authors:** 


### *Questionable Advertisements.*

Under the above head, the (Philadelphia) American Presbyterian has a most excellent article, condemning the practice of admitting into the columns of public prints those objectionable medical advertisements with which our papers are flooded, and which are so injurious, both to body and mind, in their tendencies. It is not a little remarkable, that our journals, all of whom profess to uphold the cause of morality, if not of decency, should generally be silent upon the subject of so great an outrage. Even the large sums which are paid for the insertion of such advertisements should not, one would think, be sufficient to silence almost the whole press, on a subject of so much interest to the welfare of the community. There is not a city, and hardly a town, in our country, in which one or more newspapers are not printed, containing habitually advertisements which, if not grossly indecent, are the most barefaced impositions. Men subscribe for journals whose columns are filled with announcements which cannot be read by their wives and daughters without feelings of shame and indignation, nor by their sons without danger.

It is not our province to point out the moral evils which inevitably follow this state of things; but in the name of the profession, in the name of humanity, we tender our thanks to the Presbyterian for its remonstrance against the practice of admitting into newspapers advertisements which hold out delusive hopes to the sick, and after inducing them to spend their money for worthless, if not pernicious compounds, leave them in a worse state than before. We are aware that our motives will be misconstrued by some; that our indignation may be prompted by the jealousy occasioned by the success of "illegitimate" medicine. The charge is simply absurd. Individuals may be occasionally injured by the success of empirics, but as we have stated before now, the profession is indirectly, and many physicians are directly benefitted by the unfortunate consequences of taking quack medicines. It is the deluded public who suffer, a large portion of whom can only be made to believe, after they have been taught by bitter experience, that ignorant pretence, unblushing impudence, barefaced imposture, are but a poor dependence in time of need. We appeal to the respectability of our profession throughout the land, throughout the world, as a proof of the purity of our motives.

The journal from which we quote copies a number of advertisements, whose absurdity would provoke laughter, if it did not excite our pity that such transparent frauds should be played off on the public, with hardly a remonstrance from the press, which, on the contrary, in too many instances strongly recommends them to the patronage of the public.

"We take up a paper," says the Presbyterian, and "as we read we find the announcement that a certain person offers his "*Cancer Drops and Ointment*" to those afflicted with cancer and scrofula. With more modesty than ordinarily characterizes the vender of such nostrums, he only asserts that it is a 'safe and generally certain remedy for such diseases.' Now, are the editors of that paper so grossly ignorant as to believe that cancer or scrofula can be cured by 'ointment and drops?' Do they not know that thousands are sent to premature graves by drugging themselves within, and plastering themselves without, with such nostrums?"

We subjoin another extract:

"But, as if this were not enough—and how strange it is that with such a remedy any other should be in demand—we have, in the same paper, a disinterested individual, who has a certain cure for consumption, which he longs to give the suffering. Hear him:

"'A retired physician, whose sands of life have nearly run out, discovered, while living in the East Indies, a certain cure for consumption, bronchitis, coughs, colds, and general debility. Wishing to do as much good as possible, he will send to such of his afflicted fellow beings as request it, this recipe, with full and explicit directions for making up and successfully using it. He requires each applicant to enclose him one shilling, three cents to be returned as postage on the recipe, and the remainder to be applied to the payment of this advertisement.'

"Think of it! Consumption cured for 'one shilling' and a postage stamp!! No wonder that the editors desire to spread the glad tidings among their thousands of readers.

"How is it with our ——friend? Is he engaged in the good work? Yes. He has a whole column, from the top to the bottom of his sheet, filled by the advertisement of *one* enterprising vender of these precious remedies. Here is a priceless balsam, proclaiming its virtues in paragraph upon paragraph of human grandiloquence. Cough, bronchitis, asthma, all fly before its wondrous powers of expulsion. Even consumption cannot stand it.

"'Before its delightful influence all chills, fevers, night sweats, blueness of nails, a hot, flushed skin, an uncertain strength, emaciation and decline—disappear like the poisonous dews of night before the glorious morning sun. This is no delusion, but a demonstrative fact, sustained by incontestable proof from all parts of the country.'

"If your difficulties lie in another quarter of the frame, you need not despond. We learn from the same source that anything, from Cholera to the bite of a rattlesnake, may be cured by a certain 'Pain Killer!'

"The editor, perceiving the desirableness of his readers not overlooking this invaluable medicine, favors them, under the head of

'CHEAP LIFE INSURANCE,'

with a short notice of it, and of another equally useful remedy, in a space lying between the call of the Rev. Mr. —— to the pastorate, and the marriages of the week!"

We heartily agree with the Presbyterian that the publishers of newspapers are responsible for what they send into the houses of their subscribers, and that the public may and should hold them accountable for the tendency of their *advertisements*, as well as for that of other portions of their sheet.—*Boston Medical and Surgical Journal and Medical Gazette.*

